# The Southern Dental Association

**Published:** 1894-10

**Authors:** 


ARTICLE VIII.

### THE SOUTHERN DENTAL ASSOCIATION.

### TWENTY-SIXTH ANNUAL MEETING.

The twenty-sixth annual meeting of this body was held at old point comfort, Virginia, beginning Thursday August 2d. The Association was called to order by the President Dr. B. Holly Smith of Baltimore, who proved to be a very efficient presiding officer. He was courteous, but firm and business was transacted with dispatch and without confusion. An address of welcome was made by Dr. E. P. Beadles, on the part of the Virginia State Dental Association, which had just adjourned. This was followed by an address of the President, in which he alluded to the value of such gatherings, their educational influence, which might be enhanced by more careful and thorough digestion of the principles to be discussed by the various committees. Each year nearly the same questions were debated without ever arriving at a definite conclusion. Some of these should be authoritively decided, and thus settled forever. One of them was the whole matter of Dental legislation, including admission of to practice of the non graduates. The address was, on motion, referred to a committee consisting of Drs. J Taft, J. Y Crawford, and B. H. Catching.

The report of the committee on Prosthetic Dentistry was presented, and Dr. T. P. Hinman read a paper entitled, "An Original Method of Protecting the Tips of Porcelain Teeth in Bridge Work, and a Simple Method of Banding a Logan Crown." His method of protecting porcelain facings from the strain of use was to bevel the porcelain on the inside upon a corundum stone, grinding toward the cutting edge, so as to leave it quite sharp. The tooth is now backed with gold, the backing being left to extend one-sixteenth of an inch beyond the cutting edge, but not lying close to it. All of the backing, except that part over the cutting edge, is then covered with wax, and it is so invested as to cover everything except the wax. After the removal of the wax, solder is flowed over it to the proper contour. The teeth are then waxed into proper position on the bridge, invested and united by solder. After cooling the projecting edges of the backing, which have not been covered by solder, are burnished down upon the teeth, and the piece is finished.

To band a Logan crown, the essayist prepares the tooth as for a Richmond, and fits to it a band of gold more than usually wide, and larger at its opening than at the root end. A crown is selected and ground approximately to fit the root. The band is then put in place and the end of the crown ground so closely to fit it, when it is driven home with a mallet. The whole is then removed and the front part of the band ground away, leaving it long on the palatal portion, which is carefully burnished down to the porcelain and over the bulbous portion, thus causing it to hold the crown firmly.

Dr Parr, of New York, said that it would be found difficult to burnish the gold down upon the porcelain tip so that it would protect it.

Dr. Kulp, of Davenport, Iowa, said that he had never been able to accomplish this satisfactorily, but had depended upon the use of platinum, carefully annealed and burnished down, which was then covered with solder.

Dr. Hinman in closing, said that he secured the adaptation of the gold to the beveled edge by rubbing it down with a piece of corundum or pumice stone.

A paper was read by Dr. E. P. Beadles, of Danville, Va., upon "Anterior Fillings," and at the evening session, another by Dr. S. W. Foster upon "Some of the Merits of Contour Fillings made in the laboratory."

The forenoon of each day after the first was taken up by clinics, at which various manipulative procedures in operative and mechanical dentistry were demonstrated. The clinicians were plentiful, and the most of the operations of interest. The members gathered about the operating chairs and mechanical benches, freely conversing and discussing, and at times criticising the different methods recommended and followed. All seemed to take a lively interest in them, which continued unabated to the end.

The heat was intense, and it sometimes became necessary, for those who came from a northern climate especially to adjourn to the cooler verandas, where the breeze from the bay could be felt, but nothing seemed to dampen the ardor of some of the southern members. On the morning succeeding each clinic, a report was made to the Association upon the methods employed, and the success which attended them.

A number of papers were read by different members some of which were of a high order of merit, others being rather too dogmatic in their assertions for ready acceptance. This is always an evidence of narrow views, of insufficient experience and restricted observation. When one becomes familiar with other methods than his own, and thoroughly comprehends basal principles, he learns that mere methods of manipulation count for very little, and that scientific laws are broad in their application.

On Friday evening, Dr. W. C. Carrett, of Buffalo, by special invitation, gave an account of the organization of the University of the State of New York, and the good work it is doing in educational matters, with the new regulations which, by authority of the Legislature, it has imposed upon professional schools of New York. He described the duties of the Regents of the State University, and their supervisory powers over all the higher educational institutional institutions of the State. He gave an account of the Regents' examinations, and the Regents' certificates of qualification in the various higher studies, and said that within the past year they had issued regulations for the matriculation of dental students, which were identical with those of medicine. Henceforth, no student could be received in a dental college within the State of New York, without the evidence of preliminary training that a Regents' certificate gives.

The Deans henceforth have nothing to do with determining the qualifications of proposed students. They have no authority, save to receive those who come armed with the Regents' certificate.

The reports of the Committees on Chemistry, on Pathology and Therapeutics, on Dental Education, on Histology and Microscopy; and on Dental Hygiene, were presented at different times, together with those on literature, on Operative Dentistry, and on Appliances and Improvements. These were in some cases, accompanied by papers, and all were enthusiastically and intelligently discussed. The members of The Southern Dental Association are more given to rhetoric than is usual in the American, and the eagle occasionally screams his loudest. Sometimes, as in other societies, there is too much talk for the mere sake of talk, and sound subdues sense, but there is never a lack of attention, nor a flagging of interest in the proceedings. Altogether, the meeting was a profitable one and the mingling of the representatives from the extremes of the country members of the Southern and the American Associations tended to broaden the views of all and to be mutually beneficial.

The place selected for the next meeting was Atlanta, Ga. The officers elected to serve for the coming year are: President, H E. Beach, Clarksville, Tenn.; first vice-president, Louis P, Dotterer, Charleston, S. C.; Third vice-president, J. W. Seals, Stuttgart Ark.; corresponding secretary, E. P. Beadles, Danville, Va.; recording secretary, S. W. Foster, Decatur, Ala.; treasurer, H. A. Lawrence, Athens, Ga.; Executive committee vacancies, S. B. Cook, Chattanooga, Tenn., V. E. Turner, Raleigh, N. C.—*Dental Practitioner and Adverttser.*

